# Socioeconomic inequalities in smoking in low and mid income countries: positive gradients among women?

**DOI:** 10.1186/1475-9276-13-14

**Published:** 2014-02-06

**Authors:** Jizzo R Bosdriesz, Selma Mehmedovic, Margot I Witvliet, Anton E Kunst

**Affiliations:** 1 Department of Public Health, Academic Medical Centre -University of Amsterdam, Amsterdam, The Netherlands

**Keywords:** Smoking, Socio-economic status, Women, Inequalities, Smoking epidemic, Global, World health survey

## Abstract

**Background:**

In Southern Europe, smoking among older women was more prevalent among the high educated than the lower educated, we call this a positive gradient. This is dominant in the early stages of the smoking epidemic model, later replaced by a negative gradient. The aim of this study is to assess if a positive gradient in smoking can also be observed in low and middle income countries in other regions of the world.

**Methods:**

We used data of the World Health Survey from 49 countries and a total of 233,917 respondents. Multilevel logistic regression was used to model associations between individual level smoking and both individual level and country level determinants. We stratified results by education, occupation, sex and generation (younger vs. older than 45). Countries were grouped based on GDP and region.

**Results:**

In Eastern Europe and the Eastern Mediterranean, we observed a positive gradient in smoking among older women and a negative gradient among younger women. In Sub-Saharan Africa and Latin America no clear gradient was observed: inequalities were relatively small. In South-East Asia and East Asia a strong negative gradient was observed. Among men, no positive gradients were observed, and like women the strongest negative gradients were seen in South-East Asia and East Asia.

**Conclusions:**

A positive socio-economic gradient in smoking was found among older women in two regions, but not among younger women. But contrary to predictions derived from the smoking epidemic model, from a worldwide perspective the positive gradients are the exception rather than the rule.

## Introduction

The number of smokers worldwide is around 1.4 billion today and is projected to reach around 1.8 billion by 2030 [1]. This results in 6-8 million smoking-attributable premature deaths per year between 2010 and 2030, with about 80% of these deaths occurring in low- and lower-middle-income countries (LLMICs) [2]. In most high income countries today, there is a negative gradient in smoking, i.e. smoking is most prevalent among low socio-economic status (SES) groups [3-5]. As a result, smoking is one of the most important contributing factors to inequalities in health.

In some Southern European countries however, until recently a positive gradient (smoking being more common among high SES groups) was observed among older women [6,7]. This could be linked to the smoking epidemic model, which describes the distribution of smoking across the population over time [8]. Although the smoking epidemic model originally did not prominently feature SES, it is mentioned that in early stages, smoking prevalence among high SES groups was similar to, or higher than among low SES groups [8]. Later on, declines in smoking prevalence rates also began earlier in high SES groups than in low SES groups [8]. However, most studies based on the smoking epidemic model to date, used data derived from high income countries [3]. Therefore the suggestion of a more general applicability of the model raises the question whether these patterns can be found in other regions of the world.

In recent years, several studies on SES inequalities in smoking in LLMICs have been published, with the first international overviews finding no indications of a positive gradient. Two studies comparing national surveys in Sub-Saharan Africa found negative gradients among both men and women [9,10]. A worldwide study found the negative gradient to be largest in those LLMICs where smoking was already relatively common [11]. Other studies in low- and middle-income countries replicated these negative gradients among men, but found no clear pattern among women [12,13]. Only one of these studies compared different age-groups, enabling inferences about the development of the tobacco epidemic over generations. They found negative socio-economic gradients in all age and sex groups. These gradients were strongest among young men (aged <40) and weakest among older women (aged >40) [11].

It may be expected that social gradients in smoking differ between world regions, as regions strongly differ in terms of culture, social stratification systems, and involvement in the global economy. The goal of this study is to assess which regions, if any, display positive socio-economic gradients in smoking similar to those found in Southern Europe. We will look at a selection of 49 countries (mostly LLMICs) from six regions. Additionally, because patterns may vary over generations, we will distinguish men and women both in younger and older generations.

## Methods

### Population and data

We used data from the World Health Survey (WHS) of the World Health Organization (WHO). The details of the survey development, fielding, response analysis and initial findings are described on the website [14]. The WHS was carried out in 71 countries across all six WHO world regions from 2002-2004. Study participants were randomly selected from a nationally representative sampling frame based on the population distribution. Face-to-face interviewing was used in most countries, and telephone interviewing in the other countries. Sampling weights have corrected for differential non-response rates.

Countries were excluded from our analyses because of the following reasons: 19 countries chose not to include the module that contained smoking status, two countries had over 20% non-response on smoking status and one country lacked information on educational status. Furthermore, all respondents under the age of 18 and those missing data on any of the included variables were omitted, resulting in an analytical sample of 233,917 respondents. The list of the 49 included countries with descriptive information can be found in Table 1 and additional information on the distribution of respondents according to educational level can be found in Additional file 1.

**Table 1:** Descriptive characteristics per country by gender showing number of respondents, age standardized smoking prevalence rates and GDP per capita in 49 countries

		**N**		**Smoking prevalence %**		**GDP per capita (US $)**	**Response rate**
		**Women**	**Men**	**Women**	**Men**		
**SUB-SAHARAN AFRICA**	Burkina Faso	2,551	2,274	10.7	25.6	890	95.6
	Chad	2,457	2,199	2.8	19.4	890	91.9
	Comoros	972	787	19.5	31.0	1,040	94.6
	Congo	1,328	1,169	3.3	19.1	2,070	79.1
	Côte d’Ivoire	1,364	1,820	3.7	21.8	1,450	96.5
	Ethiopia	2,546	2,392	0.7	9.3	500	96.2
	Ghana	2,164	1,774	2.0	15.1	950	69.6
	Kenya	2,547	1,870	3.1	27.5	1,190	82.3
	Malawi	3,091	2,213	6.0	27.6	550	92.7
	Mali	1,818	2,427	4.7	25.5	810	78.7
	Mauritania	2,331	1,481	4.3	24.7	1,610	97.8
	Namibia	2,526	1,724	15.2	32.2	3,870	91.3
	Senegal	1,537	1,665	2.1	23.7	1,270	88.4
	South Africa	1,236	1,115	14.4	41.7	6,970	89.2
	Zambia	2,090	1,722	6.9	27.3	940	NA
	Zimbabwe	2,608	1,492	3.9	29.2	2,386	94.4
**LATIN AMERICA**	Brazil	2,812	2,188	18.9	28.6	7,150	100.0
	Dominican Republic	2,430	2,104	16.3	22.6	4,370	73.8
	Ecuador	2,603	2,054	7.8	31.8	5,150	77.4
	Guatemala	2,939	1,831	3.7	24.2	4,460	97.6
	Mexico	22,369	16,377	14.3	36.1	9,800	96.9
	Paraguay	2,789	2,354	15.4	45.8	3,390	97.1
	Uruguay	1,530	1,449	26.8	37.2	6,950	99.7
**EASTERN EUROPE**	Bosnia Herzegovina	594	434	27.4	53.5	5,160	93.6
	Croatia	589	401	19.8	32.3	10,430	99.7
	Czech Republic	516	419	22.4	37.9	15,560	48.8
	Estonia	645	367	23.2	53.3	11,260	99.1
	Georgia	1,590	1,165	5.1	55.3	2,480	92.4
	Hungary	828	591	27.9	38.3	12,980	100.0
	Kazakhstan	2,952	1,544	6.4	51.5	5,950	99.9
	Latvia	570	286	20.0	56.8	9,350	92.1
	Russia	2,829	1,593	10.8	56.0	8,650	99.9
	Slovakia	1,545	969	29.3	46.7	12,390	99.2
	Slovenia	314	271	18.2	26.9	18,600	44.3
	Ukraine	1,847	1,003	10.2	51.3	3,940	99.3
**EASTERN MEDITERRANEAN**	Morocco	2,926	2,074	0.5	32.8	2,900	79.4
	Pakistan	2,812	3,567	6.7	35.2	1,800	93.4
	Tunisia	2,725	2,344	2.9	51.5	4,950	95.6
	United Arab Emirates	563	617	3.6	30.3	26,750	99.7
**SOUTH-EAST ASIA**	Bangladesh	2,968	2,584	31.3	63.8	920	85.4
	India	5,145	4,849	18.3	54.2	1,660	93.0
	Myanmar	3,335	2,551	19.1	50.9	510	97.3
	Nepal	4,990	3,698	32.3	61.5	840	98.3
	Sri Lanka	3,596	3,136	3.0	40.3	2,720	98.7
**EAST ASIA**	China	2,039	1,954	4.2	59.3	2,850	99.8
	Lao PDR	2,594	2,295	16.5	68.8	1,400	97.8
	Malaysia	3,367	2,637	2.7	51.2	8,960	80.2
	Philippines	5,417	4,661	13.2	59.6	2,640	99.9
	Vietnam	1,920	1,572	2.7	54.9	1,620	83.7
	**TOTAL**	**129,854**	**104,063**	**11.8**	**39.6**	2,850*	**93.4**

*Median.

### Variables

The outcome variable was self-reported smoking status, assessed with the question: “Do you currently smoke any tobacco products such as cigarettes, cigars, or pipes?” Responses ‘Daily’ and ‘Yes but not daily’ were combined into ‘smokers’ , and ‘No, not at all’ was used as the reference group.

The included individual-level covariates were sex, age, highest completed level of education and current occupational status. Age ranged from 18 upwards, grouped into seven categories (18-25, 26-35, 36-45, 46-55, 56-65, 66-75 and 76+). For the analyses by generation, we dichotomized age in younger (18-44) and older (45+). Educational level was measured in five categories: (I) less than primary school, (II) primary school, (III) secondary school, (IV) high school and (V) university or higher. Note that some of those aged 18-25 years have not yet completed their education; they were classified according to their current education. Current occupational status was assessed using eight categories, ranging from ‘not working for pay’ to a combined group of all ‘white collar’ classes.

Socio-economic position was also assessed on the country level by Gross Domestic Product per capita (GDP). GDP per capita was obtained from the WHO 2002 figures, with the exception of Zimbabwe, which was taken from the 2002 United Nations Statistics Division records [15,16]. In a sensitivity analysis, female literacy rate was used instead and this produced largely similar results as those presented below. Countries were grouped into low- and lower-middle income-countries (LLMICs) where GDP <3,856 US$, and upper-middle and high-income-countries (UMHICs) where GDP ≥3,856, based on definitions of the 2008 World Bank income categories [17].

To perform analyses per region, we grouped the countries based on the organizational regions of the WHO [18]. Because we only included certain countries within these regions, we have renamed four regions to more accurately describe the selection of countries. From the WHO’s terminology, Africa became Sub-Saharan Africa, the Americas became Latin America, Europe became Eastern Europe and the Western Pacific became East Asia. The names Eastern Mediterranean and South-East Asia were retained.

### Analysis

We calculated age standardised smoking prevalence rates per sex and educational level, by means of the direct method and using the WHO’s world standard population [19]. Since smoking rates differ greatly by gender, especially in LLMICs [20], all further analyses were stratified by gender.

To investigate whether smoking is associated with individual level SES indicators, we fit two-level logistic models with a random intercept to the hierarchical data of individual respondents nested within countries. The multilevel logistic models allowed for the expected clustering of outcomes among individuals from the same country. All models included age, education and occupation as independent variables while adjusting for country-level GDP. In a second analysis, we additionally stratified by generation. For all analyses, odds ratios (OR’s) and 95% confidence intervals (95% CI’s) were calculated. All analyses were conducted in Stata (version 9.2).

## Results

Descriptive country information is provided in Table 1. There were slightly more women than men in the overall sample (55.5% and 44.5% respectively). Smoking prevalence rates among women were low in the Sub-Saharan Africa, Eastern Mediterranean and East Asia regions (<10%), and relatively high in Eastern Europe, Latin America and Southeast Asia (15%-21%). Among men, the lowest prevalence rates were found in Sub-Saharan Africa (25%) and the highest in East Asia (55%).

Table 2 shows that in both LLMICs and UMHICs, men and women in white collar functions consistently smoke less than those in other occupational groups. Furthermore, for both women and men, the higher education groups have lower odds of smoking than the lowest education group. This difference is slightly larger in LLMICs for men and significantly larger in LLMICs for women.

**Table 2:** Associations between smoking, age and socio-economic factors, per national income level, for women and men

		**Odds ratio (95% CI)**			
		**Women**		**Men**	
		LLMIC*	UMHIC**	LLMIC*	UMHIC**
**AGE**	18 - 25	1.00	1.00	1.00	1.00
	26 - 35	1.68 (1.52-1.85)	1.07 (0.99-1.16)	1.89 (1.78-2.00)	1.22 (1.14-1.31)
	36 - 45	2.71 (2.46-3.00)	1.14 (1.05-1.24)	2.34 (2.21-2.48)	1.27 (1.19-1.36)
	46 - 55	3.84 (3.47-4.27)	1.12 (1.03-1.22)	2.28 (2.14-2.43)	1.16 (1.08-1.25)
	56 - 65	4.30 (3.84-4.82)	0.91 (0.82-1.01)	1.83 (1.70-1.97)	1.01 (0.93-1.10)
	66 - 75	4.53 (4.00-5.15)	0.67 (0.60-0.76)	1.41 (1.30-1.54)	0.78 (0.71-0.86)
	75+	4.68 (3.92-5.58)	0.60 ( 0.51-0.71)	1.28 (1.00-1.28)	0.56 (0.49-0.63)
**OCCUPATION**	White collar	1.00	1.00	1.00	1.00
	Clerk	1.17(0.80-1.17)	1.25 (1.10-1.42)	1.13 (0.98-1.30)	1.13 (1.00-1.26)
	Service or sales	1.24 (0.96-1.62)	1.44 (1.28-1.63)	1.24 (1.12-1.37)	1.18 (1.07-1.30)
	Craft trades worker	1.11 (0.84-1.47)	1.21 (1.05-1.42)	1.44 (1.30-1.58)	1.09 (1.14-1.36)
	Plant operator	1.27 (0.88-1.83)	1.42 (1.18-1.72)	1.50 (1.34-1.67)	1.24 (1.18-1.42)
	Elementary worker	1.44 (1.13-1.84)	1.27 (1.08-1.43)	1.60 (1.44-1.78)	1.30 (1.10-1.36)
	Agricultural	1.61 (1.28-2.03)	1.08 (0.88-1.35)	1.48 (1.36-1.61)	1.23 (1.00-1.19)
	Not working for pay	1.05 (0.84-1.31)	0.86 (0.78-0.95)	1.06 (0.97-1.16)	1.05 (0.97-1.14)
**EDUCATION**	No education	1.00	1.00	1.00	1.00
	Primary school	0.48 (0.44-0.52)	0.56 (0.50-0.62)	0.80 (0.77-0.85)	0.78 (0.72-0.84)
	Secondary school	0.32 (0.28-0.37)	0.63 (0.57-0.70)	0.63 (0.60-0.66)	0.72 (0.66-0.78)
	High school	0.23 (0.18-0.28)	0.77 (0.70-0.86)	0.50 (0.47-0.54)	0.67 (0.61-0.73)
	University	0.36 (0.30-0.43)	0.70 (0.61-0.80)	0.38 (0.35-0.42)	0.47 (0.42-0.52)

*LLMICs are low- and lower-middle-income countries (GDP < 3,856 dollar per capita).

**UMHICs are upper-middle- and high-income countries (GDP ≥ 3,856 dollar per capita).

Table 3 shows results stratified by generation (18-44 vs. 45+). Generally among both generations, the odds of smoking are higher for the low education groups, but among older men in UMHICs, differences between education groups are relatively small. Only among older women in UMHICs can a positive socio-economic gradient be seen.

**Table 3:** Associations between smoking and education, by national income level age group and sex

		**Odds ratio (95% CI)**			
		**Women**		**Men**	
		LLMIC*	UMHIC**	LLMIC*	UMHIC**
**Age 18-44**	No education	1.00	1.00	1.00	1.00
	Primary school	0.42 ( 0.38-0.46)	0.50 (0.43-0.57)	0.76 (0.71-0.80)	0.90 (0.83-1.00)
	Secondary school	0.26 ( 0.22-0.30)	0.29 (0.23-0.37)	0.59 (0.54-0.63)	0.70 (0.63-0.77)
	High school	0.17 (0.13-0.22)	0.21 (0.14-0.32)	0.45 (0.41-0.49)	0.55 (0.48-0.64)
	University	0.32 (0.26-0.40)	0.23 (0.16-0.32)	0.36 (0.33-0.41)	0.41 (0.35-0.47)
**Age 45+**	No education	1.00	1.00	1.00	1.00
	Primary school	0.67 (0.57-0.78)	0.65 (0.55-0.76)	0.76 (0.68-0.86)	0.86 (0.76-0.97)
	Secondary school	0.65 (0.56-0.76)	0.92 (0.78-1.08)	0.67 (0.59-0.75)	0.92 (0.81-1.04)
	High school	0.79 (0.67-0.92)	1.34 (1.12-1.60)	0.57 (0.51-0.65)	0.90 (0.78-1.03)
	University	0.57 (0.48-0.69)	1.41 (1.15-1.72)	0.37 (0.32-0.42)	0.59 (0.50-0.70)

*LLMICs are low- and lower-middle-income-countries (GDP < 3,856 dollar per capita).

**UMHICs are upper-middle- and high-income-countries (GDP ≥ 3,856 dollar per capita).

**Figure 1:**
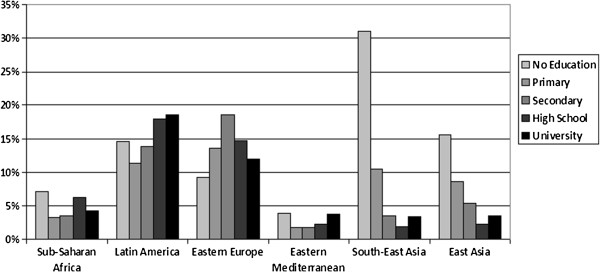
Age standardised prevalence of current smoking for women by education.

**Figure 2:**
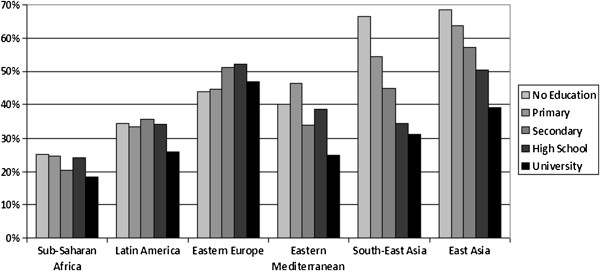
Age standardised prevalence of current smoking for men by education.

The smoking prevalence in each region, stratified by educational level, is shown for women in Figure 1 and for men in Figure 2. Educational inequalities in smoking follow markedly different patterns in different regions. In Sub-Saharan Africa and Latin America the differences between the educational groups are quite small among both men and women. Among women in Latin America and the Eastern Mediterranean a positive gradient is observed, while in South-East Asia and East Asia a strong negative gradient is visible. Among men, a slight positive gradient is observed in Eastern Europe and negative gradients in the other regions.

Table 4 presents the results for the six regions stratified by sex and according to age group. A positive gradient is observed among older women in Eastern Europe and the Eastern Mediterranean, while in the other regions, the odds of smoking are higher among the lowest education group. Among men across all regions, there is a negative gradient in both generations.

**Table 4:** Associations between smoking and education, per region and age group for women and men

		Odds ratio (95% CI)*					
	**Women**	**Sub-Saharan Africa**	**Latin America**	**Eastern Europe**	**Eastern Mediterranean**	**South-East Asia**	**East Asia**
**Age 18-44**	No education	1.00	1.00	1.00	1.00	1.00	1.00
	Primary school	0.60 (0.50-0.71)	0.70(0.56-0.88)	0.61(0.27-1.37)	1.00(0.60-1.71)	0.44(0.38-0.51)	0.43(0.35-0.53)
	Secondary school	0.58 (0.48-0.70)	0.69(0.52-0.90)	0.54(0.25-1.17)	0.64(0.30-1.40)	0.20(0.15-0.27)	0.32(0.26-0.41)
	High school	0.78 (0.65-0.95)	0.64(0.41-0.98)	0.37(0.17-0.81)	0.84(0.41-1.67)	0.11(0.07-0.20)	0.27(0.18-0.41)
	University	0.58 (0.45-0.76)	0.65(0.39-1.08)	0.36(0.16-0.80)	0.84(0.43-1.66)	0.17(0.10-0.29)	0.37(0.25-0.55)
**Age 45+**	No education	1.00	1.00	1.00	1.00	1.00	1.00
	Primary school	0.53(0.44-0.65)	0.38(0.27-0.53)	1.04(0.69-1.58)	0.63(0.24-1.64)	0.54(0.43-0.67)	0.56(0.45-0.71)
	Secondary school	0.67(0.54-0.83)	0.33(0.18-0.61)	1.44(0.97-2.14)	0.88(0.20-3.84)	0.21(0.14-0.33)	0.38(0.27-0.54)
	High school	0.84(0.66-1.08)	1.12(0.58-2.19)	1.80(1.19-2.73)	1.63(0.65-4.15)	0.11(0.04-0.30)	0.37(0.19-0.73)
	University	0.99(0.71-1.36)	0.26(0.07-0.93)	2.07(1.34-3.19)	4.53(1.66-12.41)	0.09(0.03-0.25)	0.09(0.04-0.23)
		**Odds ratio (95% CI)**					
	**Men**	**Sub-Saharan Africa**	**Latin America**	**Eastern Europe**	**Eastern Mediterranean**	**South-East Asia**	**East Asia**
**Age 18-44**	No education	1.00	1.00	1.00	1.00	1.00	1.00
	Primary school	0.58(0.50-0.66)	0.90(0.81-1.00)	1.40(0.51-3.86)	1.24(1.07-1.44)	0.72(0.64-0.80)	0.74(0.63-0.86)
	Secondary school	0.52(0.45-0.60)	0.78(0.69-0.88)	0.86(0.33-2.27)	0.71(0.60-0.85)	0.59(0.52-0.67)	0.53(0.45-0.62)
	High school	0.47(0.40-0.56)	0.80(0.67-0.95)	0.57(0.21-1.50)	0.88(0.73-1.05)	0.40(0.34-0.47)	0.39(0.33-0.47)
	University	0.40(0.32-0.50)	0.53(0.43-0.65)	0.44(0.17-1.18)	0.49(0.39-0.61)	0.28(0.23-0.47)	0.27(0.22-0.33)
**Age 45+**	No education	1.00	1.00	1.00	1.00	1.00	1.00
	Primary school	0.75(0.64-0.87)	0.96(0.81-1.13)	0.73(0.51-1.05)	1.25(1.00-1.59)	0.78(0.67-0.92)	0.73(0.60-0.86)
	Secondary school	0.76(0.64-0.91)	0.74(0.57-0.97)	0.85(0.61-1.20)	0.88(0.62-1.24)	0.64(0.53-0.77)	0.59(0.48-0.71)
	High school	0.74(0.60-0.92)	1.05(0.71-1.54)	0.81(0.57-1.14)	0.98(0.70-1.36)	0.44(0.34-0.57)	0.47(0.36-0.61)
	University	0.78(0.35-0.66)	0.60(0.42-0.85)	0.61(0.43-0.87)	0.66(0.44-0.98)	0.30(0.22-0.40)	0.39(0.30-0.52)

*All models are adjusted for individual-level age and occupation and country-level GDP.

## Discussion

Our study shows that a positive gradient in smoking is only found among older women in Eastern Europe and the Eastern Mediterranean. A strong negative gradient was observed among women in South-East Asia and East Asia, while inequalities were relatively small among women in Sub-Saharan Africa and Latin America. Among men, we did not find a significant positive gradient in any region. Inequalities were small in Sub-Saharan Africa, Latin America and Eastern Europe and negative gradients were observed in the Eastern Mediterranean, South-East Asia and East Asia.

### Limitations

The WHS has important advantages, such as providing nationally representative data for many LLMICs with a unified methodology [21], but also some limitations. For one, respondents are questioned on *current* tobacco smoking only. Unfortunately, no information on past smoking and smoking initiation is included in the survey. This information would give more insight into changing of patterns over time.

Additionally, smokeless tobacco-use, such as chewed tobacco is not assessed, although this is highly prevalent in some included countries [22]. In South-East Asian countries, where women tend to use smokeless forms of tobacco, rather than smoke [23], the smoking prevalence may be a serious underestimation of their tobacco use. This is not as much the case among men, where dual use of smokeless tobacco and smoking is much more common [23]. A study examining India found that chewed tobacco use was more common among low education and low income groups [24]. Therefore, if smokeless tobacco use had been included in the measure, educational gradients observed in South-East Asia would likely have been stronger. Future studies should measure the different forms of tobacco use, instead of assessing smoking tobacco only.

Tobacco use in this study is, like in most other studies, measured by self-reports, which might cause an underestimation of actual tobacco use [25]. There are indications that underestimation of smoking differs across educational levels in some, but not all countries [26-29]. Because of the heterogeneity in this differential underestimation across countries, it is hard to gauge what effect this could have had on our results.

Response rates of the WHS are reported to be fairly high (over 80%), but detailed information regarding the response rates of the WHS per country is not available from the original source. We have included in table 1 the figures presented by Pampel and Denney [11]. If non-response was higher for a particular sex, age, educational group, occupational group or region, our results might give an over- or underestimation of true socio-economic gradients in smoking.

### Interpretation of results

In four out of six regions, we found negative gradients in the smoking prevalence among older women and only in two regions did we find a positive gradient. Other international overview studies that addressed SES inequalities in smoking using WHS data also found mostly negative gradients, but positive gradients among older women in some countries [11-13,30]. One study using the Global Adult Tobacco Survey found significant negative gradients in most countries and no significant gradients in the remaining countries [31]. National studies found a positive gradient among older women and a negative gradient among men in Colombia and Turkey [32,33]; a positive gradient among men in Uzbekistan [34]; and negative gradients among both men and women in India [35]. Most of these results agree with our results for those regions, except that we did not find a positive gradient in Latin America.

There are several mechanisms that can have contributed to the negative socio-economic gradient in smoking that we observed in most regions. One possibility is that awareness and concern with the harm of tobacco is not equally distributed among low- and high- SES groups in LMMICs, resulting in more inequality in smoking prevalence between low- and high- SES groups [36]. A study on the contribution of various factors to the socio-economic gradient in health behaviours [37] estimated the contribution of knowledge and cognitive abilities at 30% and income (including health insurance) also at 30%. In addition, stress, self-efficacy and social support may be important mediators of the effect of SES on smoking cessation [38].

In addition, tobacco control policies are likely to have influenced the difference between regions. In most middle- and low-income countries, tobacco control policies are minimal, if present at all [39], even after introduction of the WHO’s Framework Convention on Tobacco Control (FCTC) in 2005 [40]. Most notably tobacco taxes have failed to keep up with rising income and price levels [41]. When taxes are lower, tobacco becomes more affordable for low SES groups. As a result, taxes did not have the inequality-reducing effect that was demonstrated for high taxes in affluent countries such as the UK [42]. Furthermore, the lack of advertising bans gives the tobacco industry the freedom to promote their product, mostly targeting low SES groups [43].

Why we find a positive gradient among older women in Eastern Europe, but not among younger women, might be explained in part by the emancipation of women. Initially, smoking in public was seen as unacceptable for women, which has caused the diffusion of smoking among women to lag roughly two decades behind that among men [44,45]. But, with growing emancipation, this resistance to female smoking gradually disappeared, first among high SES groups and later among low SES groups. This is similar to the developments observed in southern Europe some years before.

For the Eastern Mediterranean, we speculate that geographic proximity and cultural similarity to Southern Europe may have contributed to the positive gradient found. Especially the high SES groups in these regions are likely to be influenced by social norms from Southern Europe, where smoking among women is acceptable and follows a positive gradient [6,7,33]. At the same time in low SES groups and rural areas social norms remain conservative and traditions and religion still play an important role [33].

### Implications

We observed a positive gradient in smoking among older women, like we hypothesized, but only in Eastern Europe and the Eastern Mediterranean. From a worldwide perspective, the positive gradient seems to be the exception rather than the rule. The distribution of smoking across SES groups follows different patterns in different regions, and is likely to be influenced by region-specific factors. The smoking epidemic model has already been ‘updated’ , adding the nuance that in developing countries the model was only descriptive of men, but not for women [46]. Our results indicate that when including socio-economic inequalities in the smoking epidemic model outside developed countries, a cautious approach is warranted.

A positive gradient in smoking has the potential to mitigate inequalities in health by more strongly affecting those with a higher socio-economic status, but this beneficial effect seems to be highly localized. The negative gradient that was found in most regions will have severe consequences for the future of global health, exacerbating existing inequalities. This will become even more important in the future, as the burden of smoking-related diseases is still very much on the rise in many low and middle income countries.

## Abbreviations

FCTC: Framework convention on tobacco control; GDP: Gross domestic product; LLMICs: Low- and lower-middle-income countries; SES: Socio-economic status; UMHICs: Upper-middle and high-income countries; WHO: World health organization; WHS: World health survey.

## Competing interests

The authors declare that they have no competing interests.

## Authors’ contributions

AK and SM designed the study, MW prepared the data, SM and MW analysed the data, JB, SM, MW and AK interpreted the data. JB and SM wrote the paper, with critical revisions from MW and AK. All authors have read and approved the final version of this paper.

## Supplementary Material

Additional file 1 Educational levels for women and men in 49 countries worldwide. Click here for file
