# Supplementary material for: Socioeconomic inequalities in smoking in low and mid income countries: positive gradients among women?
Source: Int J Equity Health. 2014 Feb 6;13:14. doi: 10.1186/1475-9276-13-14 (PMC3922442; doi:10.1186/1475-9276-13-14)
Supplement: Additional file 1 — Educational levels for women and men in 49 countries worldwide. [file 1475-9276-13-14-S1.doc]

**Additional file 1: Table S1:** Educational levels for women and men in 49 countries worldwide

|  |  | **No education (%)** | | **Primary School (%)** | | **Secondary school (%)** | | **High school (%)** | | **University (%)** | |
| --- | --- | --- | --- | --- | --- | --- | --- | --- | --- | --- | --- |
|  |  | **Men** | **Women** | **Men** | **Women** | **Men** | **Women** | **Men** | **Women** | **Men** | **Women** |
| **SUB-SAHARAN AFRICA** | Burkina Faso | 88 | 81 | 6 | 8 | 4 | 6 | 1 | 2 | 1 | 2 |
|  | Chad | 94 | 80 | 3 | 8 | 2 | 6 | 1 | 1 | 0 | 2 |
|  | Côte d'Ivoire | 64 | 47 | 18 | 18 | 9 | 18 | 4 | 7 | 4 | 9 |
|  | Congo | 32 | 23 | 34 | 25 | 23 | 26 | 8 | 13 | 4 | 12 |
|  | Comoros | 77 | 63 | 11 | 15 | 8 | 14 | 1 | 2 | 2 | 6 |
|  | Ethiopia | 74 | 56 | 10 | 16 | 14 | 24 | 1 | 3 | 0 | 1 |
|  | Ghana | 52 | 35 | 40 | 50 | 5 | 8 | 1 | 2 | 1 | 5 |
|  | Kenya | 50 | 34 | 23 | 27 | 10 | 12 | 11 | 16 | 6 | 11 |
|  | Mali | 93 | 88 | 3 | 5 | 1 | 3 | 1 | 2 | 0 | 1 |
|  | Mauritania | 80 | 65 | 9 | 9 | 4 | 10 | 5 | 9 | 1 | 7 |
|  | Malawi | 80 | 63 | 16 | 26 | 4 | 9 | 0 | 0 | 1 | 2 |
|  | Namibia | 43 | 44 | 33 | 30 | 20 | 21 | 0 | 0 | 4 | 5 |
|  | Senegal | 76 | 62 | 12 | 16 | 7 | 11 | 2 | 4 | 2 | 6 |
|  | South Africa | 24 | 20 | 21 | 21 | 22 | 23 | 20 | 21 | 13 | 16 |
|  | Zambia | 52 | 36 | 38 | 46 | 6 | 11 | 1 | 1 | 3 | 6 |
|  | Zimbabwe | 34 | 21 | 37 | 34 | 26 | 36 | 1 | 4 | 2 | 5 |
| **LATIN AMERICA** | Brazil | 29 | 29 | 28 | 27 | 14 | 16 | 21 | 21 | 7 | 6 |
|  | Dominican Rep. | 61 | 65 | 19 | 20 | 15 | 11 | 1 | 0 | 5 | 4 |
|  | Ecuador | 21 | 19 | 42 | 44 | 25 | 23 | 2 | 1 | 10 | 12 |
|  | Guatemala | 66 | 61 | 12 | 12 | 7 | 9 | 7 | 8 | 4 | 5 |
|  | Mexico | 0 | 0 | 15 | 13 | 64 | 63 | 21 | 24 | 0 | 1 |
|  | Paraguay | 40 | 40 | 37 | 40 | 10 | 10 | 5 | 3 | 7 | 7 |
|  | Uruguay | 10 | 13 | 2 | 37 | 26 | 24 | 8 | 8 | 26 | 19 |
| **EASTERN EUROPE** | Bosnia Herzegovina | 29 | 12 | 25 | 17 | 39 | 61 | 4 | 5 | 3 | 5 |
|  | Croatia | 24 | 14 | 18 | 15 | 42 | 57 | 7 | 7 | 8 | 8 |
|  | Czech Republic | 1 | 0 | 20 | 12 | 32 | 41 | 37 | 34 | 10 | 13 |
|  | Estonia | 6 | 9 | 13 | 19 | 4 | 4 | 57 | 54 | 21 | 14 |
|  | Georgia | 3 | 2 | 4 | 3 | 5 | 6 | 46 | 47 | 42 | 42 |
|  | Hungary | 2 | 1 | 10 | 6 | 32 | 21 | 44 | 60 | 12 | 12 |
|  | Kazakhstan | 1 | 0 | 1 | 1 | 3 | 4 | 52 | 50 | 44 | 45 |
|  | Latvia | 7 | 5 | 17 | 26 | 43 | 36 | 20 | 21 | 14 | 13 |
|  | Russia | 4 | 1 | 6 | 4 | 29 | 31 | 20 | 21 | 41 | 42 |
|  | Slovakia | 0 | 0 | 7 | 3 | 15 | 17 | 45 | 30 | 12 | 12 |
|  | Slovenia | 5 | 1 | 25 | 15 | 16 | 31 | 36 | 38 | 19 | 14 |
|  | Ukraine | 3 | 2 | 3 | 3 | 8 | 7 | 49 | 51 | 37 | 38 |
| **EASTERN MED.*** | Morocco | 66 | 42 | 16 | 27 | 7 | 12 | 6 | 11 | 4 | 8 |
|  | Pakistan | 76 | 48 | 9 | 17 | 7 | 16 | 4 | 9 | 4 | 10 |
|  | Tunisia | 48 | 26 | 25 | 30 | 6 | 9 | 15 | 23 | 6 | 11 |
|  | Un. Arab Emirates | 20 | 12 | 6 | 6 | 11 | 15 | 19 | 1 | 44 | 50 |
| **SOUTH-EAST ASIA** | Bangladesh | 65 | 53 | 25 | 25 | 5 | 10 | 3 | 6 | 2 | 6 |
|  | India | 60 | 36 | 15 | 20 | 11 | 16 | 6 | 14 | 7 | 15 |
|  | Sri Lanka | 16 | 13 | 26 | 26 | 38 | 39 | 17 | 20 | 18 | 3 |
|  | Myanmar | 52 | 40 | 27 | 33 | 11 | 17 | 4 | 5 | 5 | 4 |
|  | Nepal | 81 | 58 | 10 | 19 | 7 | 15 | 2 | 5 | 1 | 4 |
| **EAST ASIA** | China | 26 | 13 | 23 | 23 | 26 | 34 | 15 | 17 | 10 | 13 |
|  | Lao PDR | 63 | 45 | 21 | 27 | 10 | 15 | 4 | 10 | 2 | 4 |
|  | Malaysia | 25 | 16 | 19 | 21 | 15 | 18 | 27 | 28 | 13 | 17 |
|  | Philippines | 17 | 20 | 31 | 31 | 35 | 33 | 5 | 6 | 12 | 10 |
|  | Vietnam | 29 | 16 | 24 | 23 | 29 | 35 | 13 | 18 | 9 | 8 |

* Eastern Mediterranean
